# From dipivaloylketene to tetraoxaadamantanes

**DOI:** 10.3762/bjoc.14.1

**Published:** 2018-01-02

**Authors:** Gert Kollenz, Curt Wentrup

**Affiliations:** 1 Institute of Chemistry, Karl-Franzens University of Graz, Heinrichstrasse 28, A-8010 Graz, Austria; 2 School of Chemistry and Molecular Biosciences, The University of Queensland, Brisbane, Queensland 4072, Australia

**Keywords:** bisdioxines, dipivaloylketene, tetraoxaadamantanes, transannular cyclization

## Abstract

Dipivaloylketene (**2**) is obtained by flash vacuum pyrolysis of furan-2,3-dione **6** and dimerizes to 1,3-dioxin-4-one **3**, which is a stable but reactive ketene. The transannular addition and rearrangement of enols formed by the addition of nucleophiles to the ketene function in **3** generates axially chiral 2,6,9-trioxabicyclo[3.3.1]nonadienes (bisdioxines) **4**. When arylamines are used as the nucleophiles under neutral conditions, decarboxylation occurs during the formation of bisdioxines **8**. However, when water or alcohols are added to **3** under acidic conditions, bisdioxine-carboxylic acids and esters **10** and **11** are obtained. Acid hydrolysis of the bisdioxines proceeds through the addition of water to a C=C double bond and results in a second transannular oxa-Michael-type reaction and generation of tetraoxaadamantanes **5**. This reaction is decarboxylative when free carboxylic acid functions are present in the bisdioxines, thus forming **21** and **22**, but carboxylic acid derivatives are preserved to yield compounds **20**, **23**, **25**, **28**, and **29**. A hydrogenolysis of the dibenzyl ester **23** yields the free dicarboxylic acid **24**. The tetraoxaadamantanes are formed in high yields (65–95%) in most cases, but the addition of water to the concave inside of the bisdioxines becomes severely hindered in cyclic derivatives, so that the 38-membered ring compound **32** requires microwave heating at 170 °C to form tetraoxaadamantane **33**, and the catenated compound **36** and calix[6]arene derivative **37** did not form tetraoxaadamantanes. The reaction mechanisms of bisdioxine and tetraoxaadamantane formation are discussed.

## Introduction

**Scheme 1:**
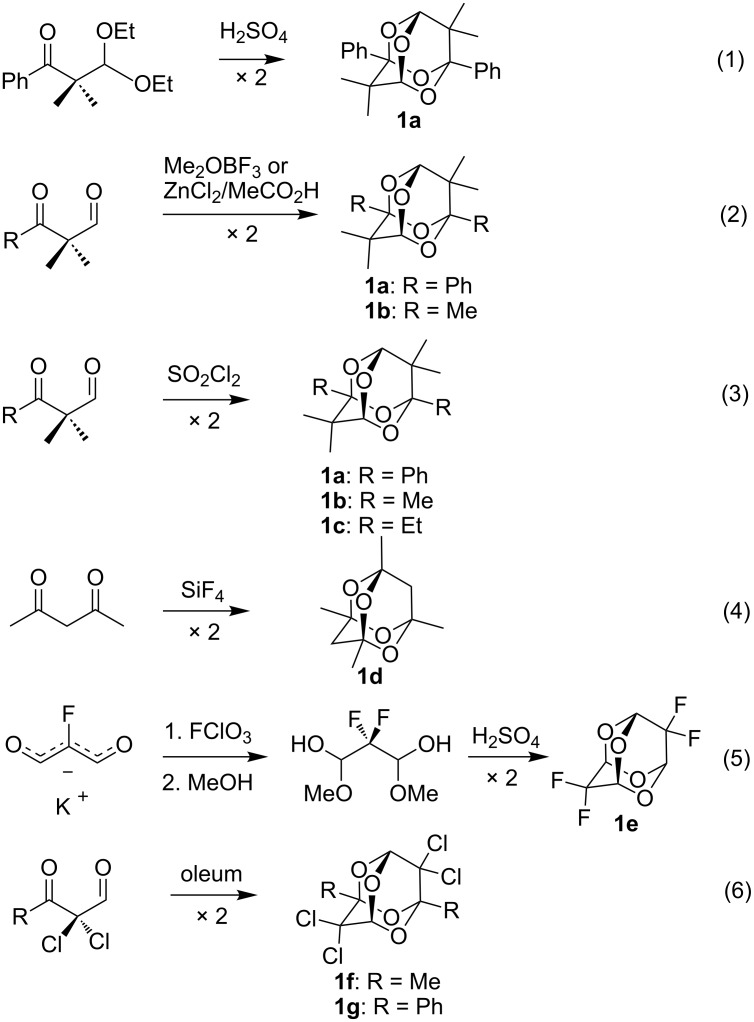
Synthetic routes to 2,4,6,8-tetraoxaadamantanes.

The tetraoxaadamantane ring system is relatively unknown and no functional group derivatives had been reported prior to our work. The first methyl and phenyl-substituted tetraoxaadamantanes **1a** and **1b** were obtained by Arnold [1] and Almqvist [2] through the dimerization of β-ketoaldehydes (reactions 1 and 2 in Scheme 1). This was confirmed by Opitz et al., but the attempted synthesis of further analogs by these procedures failed [3].

Takeda et al. performed the dimerization of β-ketoaldehydes with SO_2_Cl_2_ (reaction 3 in Scheme 1) [4], and the X-ray crystal structure of **1b** was reported [5]. Chekalov et al. described the preparation of the tetramethyl derivative **1d** by dimerization of acetylacetone induced by SiF_4_ (reaction 4 in Scheme 1) [6] and also reported the compound’s X-ray crystal structure. This transformation can also be achieved with MoOCl_4_ [7]. Dersch and Reichardt obtained the tetrafluoro derivative **1e** in the attempted preparation of difluoromalonic dialdehyde (Scheme 1, reaction 5) [8], and Guseinov et al. reported the synthesis of the corresponding tetrachlorotetraoxaadamantanes together with an X-ray crystal structure of the diphenyltetrachloro derivative **1g** (reaction 6 in Scheme 1) [9]. A tetraarsatetraoxaadamantane [10] also has been reported, and ^13^C NMR spectral characteristics of tetraoxa-, tetrathia-, and tetraselenaadamantanes have been discussed [11], but due to the lack of functional groups, tetraoxaadamantanes have remained largely laboratory curiosities.

It is worth noting that the 2,4,10-trioxaadamantanes, which are orthoesters, are well known [12–13], and the natural product muamvatin [14] is a 2,6,9-trioxaadamantane derivative. The dioxaadamantanes are best known in the form of the highly toxic tetrodotoxins occurring in the puffer fish, certain newt species and several other aquatic animals [15].

**Scheme 2:**
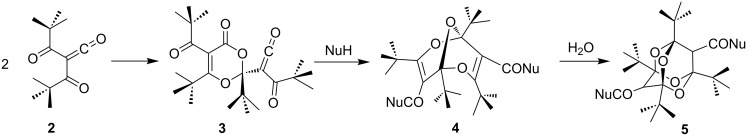
Conversion of dipivaloylketene (**2**) to bisdioxines (2,6,9-trioxabicyclo[3.3.1]nona-3,7-dienes) **4** and tetraoxaadamantanes **5**.

In our laboratories we have developed an efficient and high-yielding synthesis of tetraoxaadamantanes **5** by employing two unusual reaction steps: (i) the conversion of the dimer **3** of dipivaloylketene (**2**) to bisdioxines (2,6,9-trioxabicyclo[3.3.1]nona-3,7-dienes) **4**, by the addition of nucleophiles, and (ii) the facile acid-catalyzed hydrolysis of **4** with concomitant transannular cyclization. Following this route a wide variety of compounds **5** containing functional groups is accessible (Scheme 2).

In this review we will start with an overview of the syntheses and chemistry of the bisdioxines leading on to the syntheses of tetraoxaadamantanes.

## Review

### Bisdioxines

**Scheme 3:**
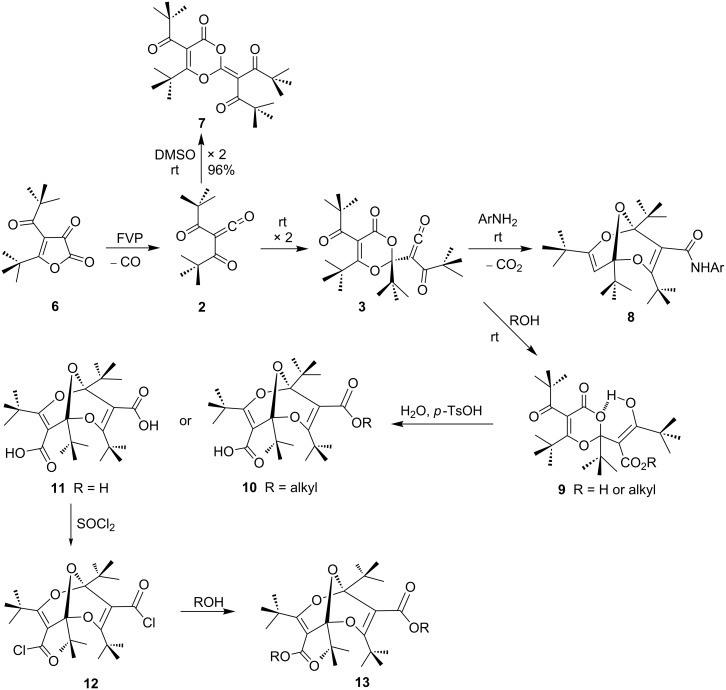
2,6,9-Trioxabicyclo[3.3.1]nonadienes (bisdioxines, **9**–**13**) derived from dipivaloylketene (**2**).

The synthesis of bisdioxines **8**–**13** starts with furandione **6**, which, upon flash vacuum pyrolysis (FVP) at 350–500 °C at 10^−3^–10^−4^ hPa, eliminates a molecule of CO to generate dipivaloylketene (**2**) in over 90% yield (Scheme 3).

Usually, α-oxoketenes are not isolable, but due to the steric hindrance exerted by the pivaloyl groups ketene **2** is kinetically stable at up to −20 °C. However, it dimerizes at room temperature to afford an 88% yield of the thermally very stable dimer **3**, which still carries a ketene function [16]. Compound **3** is formed through a [2 + 4] cycloaddition between one molecule of the α-oxoketene **2** and the carbonyl C=O bond of a second molecule. It is noteworthy that in the presence of DMSO a different dimer **7** is formed, again in high yield, originating from a [2 + 4] cycloaddition between a molecule of the α-oxoketene and the ketene C=O bond of the second molecule (Scheme 3) [17].

**Scheme 4:**
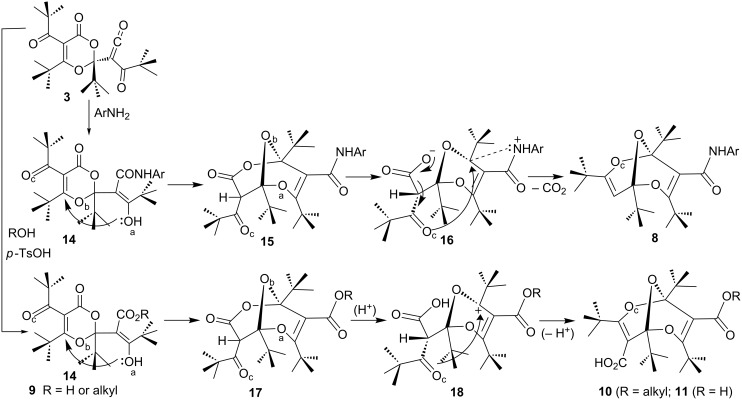
Mechanisms of formation of bisdioxine acid derivatives from dimer **3**.

The treatment of the dimeric ketene **3** with nucleophiles allowed the preparation of numerous derivatives of the unique 2,6,9-trioxabicyclo[3.3.1]nonadiene (bisdioxine) system **8**–**13**, namely the monoamides **8**, the diacid **11**, the diacid dichloride **12**, and the esters **9**, **10** and **13** [18–19] (Scheme 3). The mechanism of formation of these derivatives is summarized in Scheme 4.

The amides **8**, formed by the addition of arylamines are invariably decarboxylated. We interpret this in terms of the sequence **3 → 14** → **15** → **16**, which in the absence of an acid decarboxylates. In contrast, the addition of water and alcohols to **3** requires the addition of acid (*p*-TsOH) and results in isolable enol intermediates of type **9** [18]. The carboxylate cannot form in the presence of an acid and therefore decarboxylation does not take place (**17** → **18** → **10**). The mono- and dicarboxylic acids **10** and **11** are stable and do not decarboxylate easily.

However, aromatic amines carrying strongly electron-withdrawing groups (nitroanilines) do not form stable bisdioxines such as **8**. Instead, a cleavage of the dioxinone ring in **3** with formation of dipivaloylacetamides takes place. Surprisingly, the more basic aliphatic amines also cause cleavage to dipivaloylacetamides. On the other hand, neutral thiols do not add to **3**, but in the presence of triethylamine ring opening again takes place [18]. Further chemistry of the ketenes obtained by FVP of 5-*tert*-butyl-4-pivaloylfurandione (**6**) and 5-*tert*-butyl*-*4-methoxyfurandione and their dimers has been reported [20].

It is worth noting that the bisdioxines exhibit axial chirality [21], as has been demonstrated by ^1^H NMR spectroscopy using the optically active shift reagent Eu(hfc)_3_ [19]. The enantiomers of the dicarboxylic acid **11** have been separated by flash chromatography of their diastereomeric salts with 1-phenethylamine, and the structures of the acids and ethyl esters were determined by X-ray crystallography [19]. The X-ray structure of the Pt(II) chelate of tetramethyl 2,6,9-trioxabicyclo[3.3.1]nona-3,7-diene obtained from tris*-*acetylacetonato platinum(II) was determined previously [22–23], and the separation of the enantiomers of the free ligand was achieved by fractional crystallization [24]. Chirality of the bisdioxine dicarbaldehyde, 2,6,9-trioxabicyclo[3.3.l]nona-3,7-diene-4,8-dicarbaldehyde, obtained by extrusion of water from triformylmethane, has also been demonstrated [25], and X-ray crystallography confirmed the structure of this molecule, too [26].

**Scheme 5:**
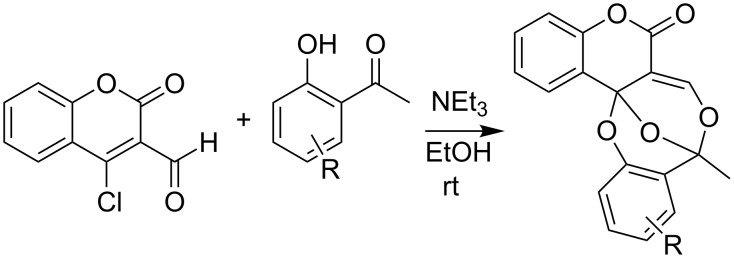
Recently reported synthesis of chromenobisdioxines.

There are few other reports of bisdioxines in the literature. The synthesis of dimethyl bisdioxinedicarboxylate has been described [27], and recently a new synthesis of chromenobisdioxines based on a mild base-mediated reaction of 4-chloro-3-formylcoumarin and *o*-hydroxyacetophenones has been reported (Scheme 5) [28].

### Formation of tetraoxaadamantanes

**Scheme 6:**
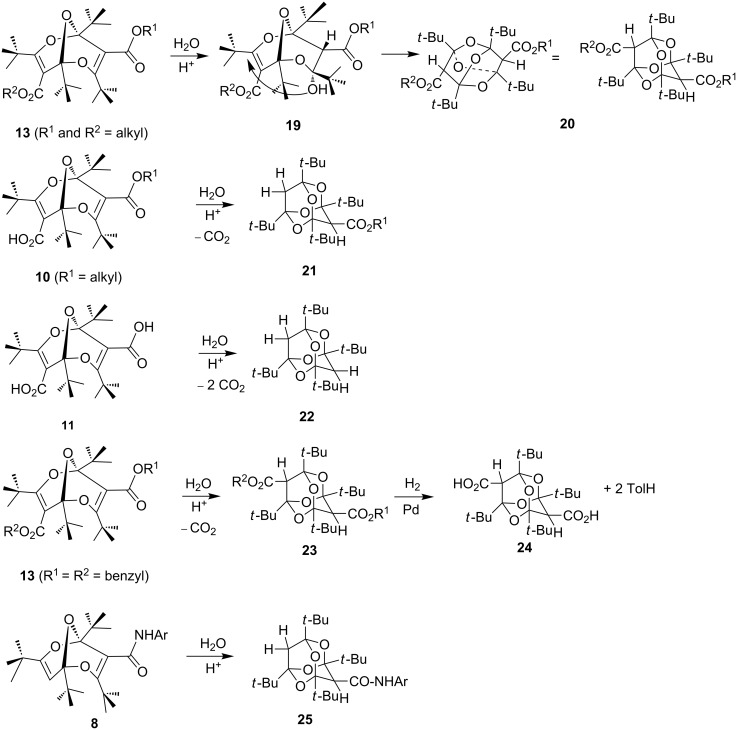
Formation of tetraoxaadamantanes.

Although the bisdioxine skeleton is a thermodynamically stable moiety, allowing numerous derivatives to be synthesized, it was soon discovered that in the presence of strong acids, very efficient addition of water and cyclization to tetraoxaadamantanes **20**–**25** took place (Scheme 6) [29].

**Scheme 7:**
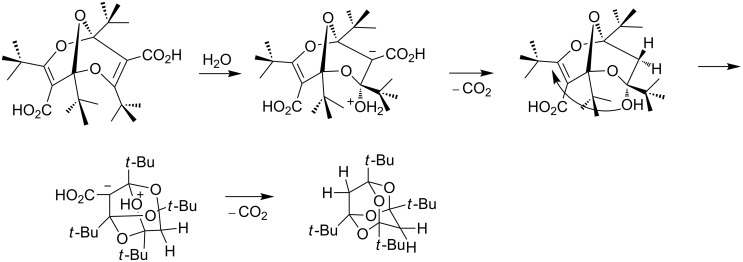
Decarboxylative hydrolysis and oxa-Michael-type ring closure.

The reaction is usually carried out at room temperature in dichloromethane in the presence of concentrated HCl and glacial acetic acid, and the yields are mostly in the range 65–95%. Depending on the starting material, diesters, monoesters or the fully decarboxylated tetraoxaadamantane can be obtained. It is noteworthy that, when a free carboxylic acid moiety is present in the bisdioxine, it is invariably lost during the tetraoxaadamantane formation. The decarboxylation is likely to take place in the acrylic acid moieties in the trioxanonadienes during the reaction (Scheme 7), and not in the final products, which are not prone to decarboxylation: the stable bis-carboxylic acid **24** can be obtained by hydrogenolysis of the dibenzyl ester **23** (Scheme 6) [30]. The reaction may be seen as a decarboxylative [31–32] oxa-Michael addition (Scheme 7) and may be related to the recently described acid-catalyzed decarboxylation of vinylic and aromatic carboxylic acids [33].

The amide derivatives **8** react in the same way as monoesters, forming arylaminotetraoxaadamantanes **25** (Scheme 6) [29,34]. The X-ray crystal structure of **25** (Ar = *p*-methoxyphenyl) has been published [29]. It should be noted that both the bisdioxines [19] and the tetraoxaadamantanes [29] exhibit axial chirality as confirmed by ^1^H NMR spectroscopy with the Eu(hfc)_3_ chiral shift reagent.

**Scheme 8:**
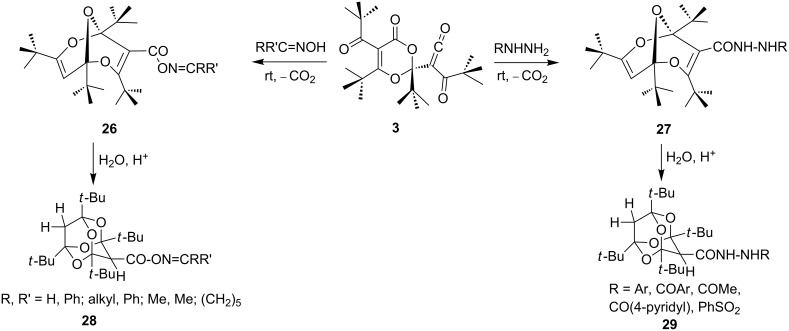
Oxime and hydrazine derivatives of bisdioxines and tetraoxaadamantanes.

Bisdioxine oxime and hydrazine derivatives **26** and **27** (Scheme 8) are formed from **3** at room temperature without the need for acid catalysis. As in the case of the addition of arylamines, monodecarboxylation takes place, and in the presence of a strong acid, they are converted to the tetraoxaadamantanes **28** and **29** (65–93%) [35].

**Figure 1:**
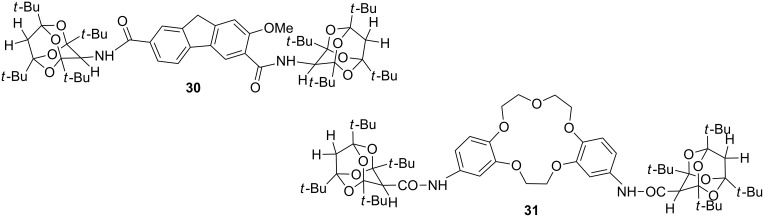
Bistetraoxaadamantane derivatives.

Several bisdioxine derivatives of aromatic di- and triamines as well as crown-ether derivatives were prepared from **3** in order to examine their host–guest properties by ESI mass spectrometry and NMR spectroscopy. Some tetraoxaadamantanes were also examined in this way. For example, compound **30** (Figure 1) was found to have a particular affinity for complexation with choline [26,36], and the crown-5 derivative **31** showed an enhanced ability to extract Na^+^ and K^+^ ions from water into CHCl_3_ (22 and 21%, respectively, within 10 minutes using equimolar amounts of salt and ligand) [37].

**Scheme 9:**
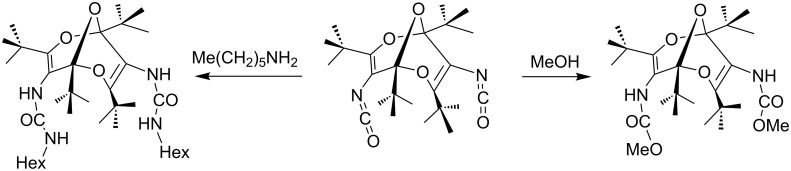
Inward-pointing isocyanate, urethane and carbamate groups in bisdioxines. The diisocyanate is obtained by Curtius and Hofmann rearrangements of the diazides and diamides [38–39].

In the concave structures of the bisdioxines, the functional groups such as esters, amides, carbamates, urethanes and isocyanates point inward (Scheme 9).

**Scheme 10:**
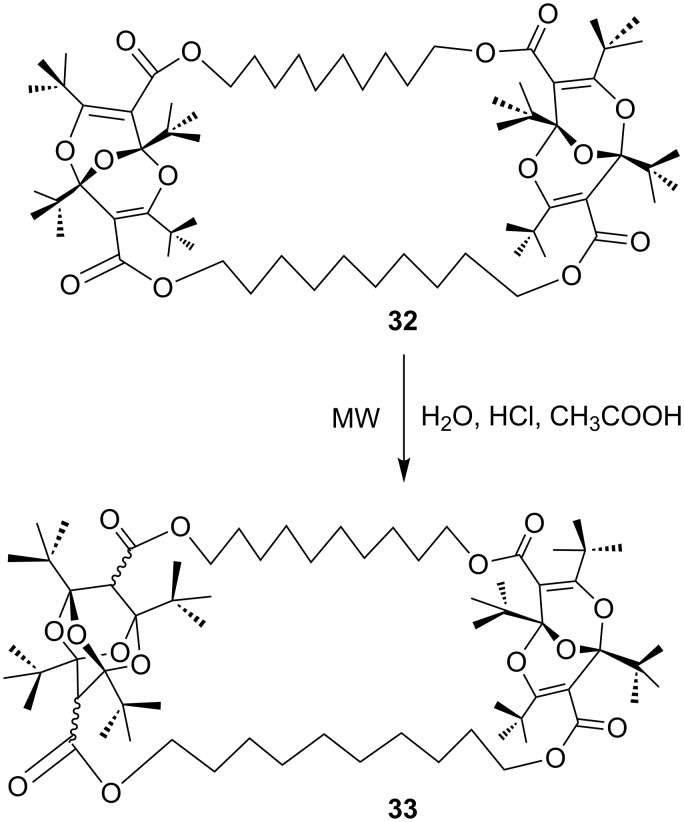
Microwave-assisted tetraoxaadamantane formation.

This observation was confirmed by X-ray crystallography as well as calculations at the B3LYP/6-31G** level [38–39]. The *tert*-butyl groups provide steric protection to the *exo* sides of the molecules, making the diisocyanate stable at ordinary temperatures. However, it readily reacts with amines and alcohols to form ureas and urethanes, respectively. Taking advantage of this type of concave structure, several cyclic derivatives were synthesized, and some of them were converted to tetraoxaadamantanes [37]. However, when two bisdioxine units are present in a cyclic structure as in Scheme 10, the formation of tetraoxaadamantanes requires the addition of water from the concave inside. Thus, the bisdioxine **32** did not form a tetraoxaadamantane **33** under the usual reaction conditions, but this was finally achieved in 35% yield by microwave irradiation at 170 °C for 40 min [40].

**Scheme 11:**
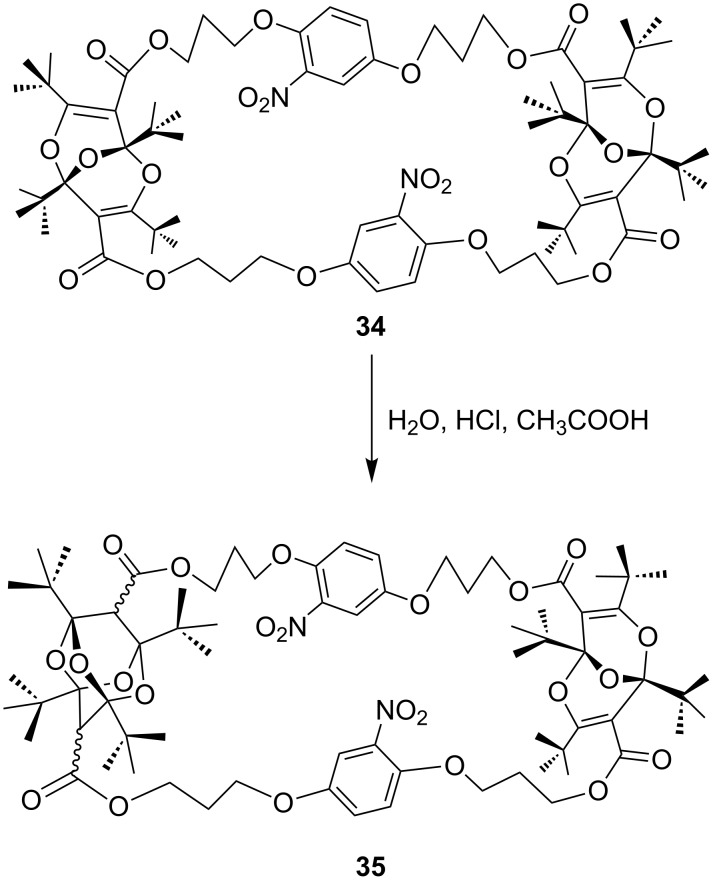
Cyclic bisdioxine ester derivative **34** forming a single mono-tetraoxaadamantane.

**Figure 2:**
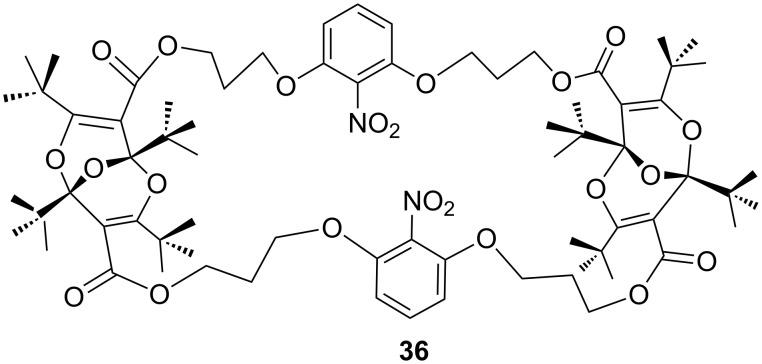
Cyclic bisdioxine derivative not forming a tetraoxaadamantane due to reduced cavity size.

This subject was investigated further by synthesizing the cyclic bisdioxine ester derivatives **34** and **36** (Scheme 11 and Figure 2) [41]. The 1,4-catenated dinitro compound **34** is readily converted to the mono-tetraoxaadamantane derivative **35** (Scheme 11). However, all attempts to convert the second bisdioxine unit were fruitless, presumably due to steric hindrance: the cavity in **34** is large enough to form one tetraoxaadamantane derivative, but this reduces the available space, so that the attack by another water molecule on the second bisdioxine unit from the concave inside in **35** was not observed. Force-field calculations indicated that the internal cavity is significantly smaller in the 1,3-catenated nitro compound **36** than in **34**, and in fact it was not possible to prepare any tetraoxaadamantane derivative from **36** (Figure 2).

**Figure 3:**
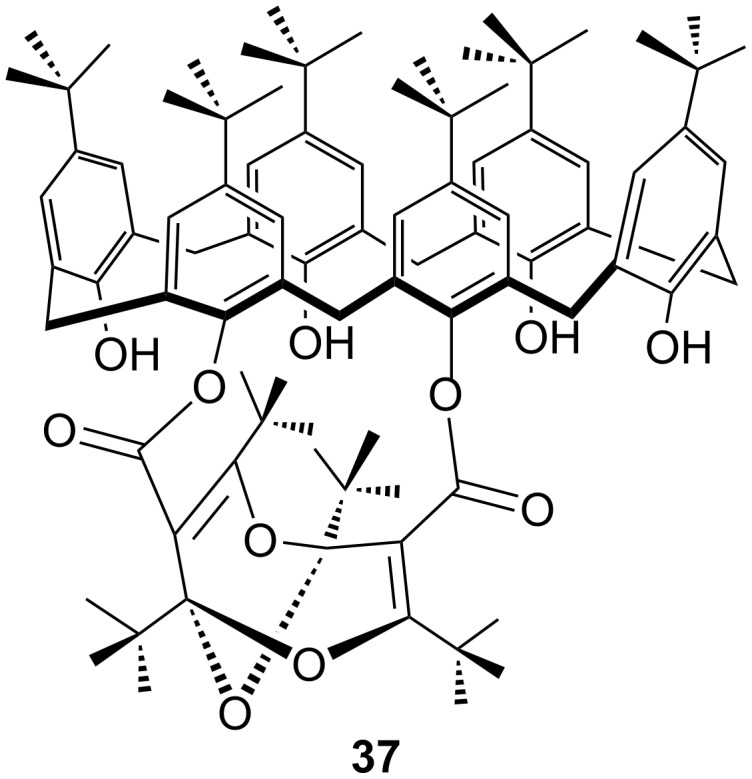
The bisdioxine-calix[6]arene derivative **37** complexes Cs^+^ but does not form a tetraoxaadamantane derivative.

An even higher hindrance is induced by the *p-tert-*butylcalix[6]arene moiety in **37** (Figure 3), which is obtained from the bisdioxine diacid dichloride **12** and calixarene [42]. The wider upper rim is clearly seen in the X-ray structure and the compound demonstrates a pronounced ability to extract Cs^+^ ions from water into chloroform by forming endohedral complexes, which is typical for capped calixarenes [43]. However, the lower rim is very congested, thereby hindering the endohedral addition of water to the bisdioxine unit, and in fact a tetraoxaadamantane derivative was not formed.

## Conclusion

The stable 1,3-dioxin-4-one ketene derivative **3** is obtained by dimerization of dipivaloylketene (**2**), itself obtained in high yield by FVP of furan-2,3-dione **6** (Scheme 3). Ketene **3** reacts with a variety of nucleophiles in an addition reaction to the ketene function, thereby transforming the ketene to enol derivatives **14** or **9** of 1,3-dioxin-4-ones. Subsequently, these enols can undergo transannular cyclizations to yield initial intermediates **15** and **17**. However, the latter compounds rearrange, whereby an O–C=O moiety in the 1,3-dioxinone becomes a carboxylic acid function in the resulting, axially chiral 2,6,9-trioxabicyclo[3.3.1]nonadienes (bisdioxines) **8**, **10**, and **11** (Scheme 4). When this reaction is carried out under neutral conditions (with arylamines), decarboxylation of the carboxylic acid function occurs, yielding **8**, but under acidic conditions (with alcohols and water) the acid function is preserved, yielding **10** and **11**.

Addition of water to one of the acrylic-type double bonds in the bisdioxines under acidic conditions generates a tertiary alcohol, which again undergoes a transannular oxa-Michael-type ring closure forming a tetraoxaadamantane. Free carboxylic acid functions are decarboxylated in this process (Scheme 7), but amide and ester functions are preserved in products **20**, **23**, **25**, **28**, and **29**. The dibenzyl ester **23** can be hydrogenated to yield the free dicarboxylic acid **24** (Scheme 6). The tetraoxaadamantane-forming reaction is very efficient and high-yielding, taking place in a variety of open-chain and catenated bisdioxine derivatives. However, the 38-membered ring **32** requires forcing conditions to form a tetraoxaadamantane, and compounds **36** and **37** did not form tetraoxaadamantanes at all.
